# Curricula for teaching end-users to kinesthetically program collaborative robots

**DOI:** 10.1371/journal.pone.0294786

**Published:** 2023-12-01

**Authors:** Gopika Ajaykumar, Gregory D. Hager, Chien-Ming Huang

**Affiliations:** Department of Computer Science, Johns Hopkins University, Baltimore, MD, United States of America; University of Namibia, NAMIBIA

## Abstract

Non-expert users can now program robots using various end-user robot programming methods, which have widened the use of robots and lowered barriers preventing robot use by laypeople. Kinesthetic teaching is a common form of end-user robot programming, allowing users to forgo writing code by physically guiding the robot to demonstrate behaviors. Although it can be more accessible than writing code, kinesthetic teaching is difficult in practice because of users’ unfamiliarity with kinematics or limitations of robots and programming interfaces. Developing good kinesthetic demonstrations requires physical and cognitive skills, such as the ability to plan effective grasps for different task objects and constraints, to overcome programming difficulties. How to help users learn these skills remains a largely unexplored question, with users conventionally learning through self-guided practice. Our study compares how self-guided practice compares with curriculum-based training in building users’ programming proficiency. While we found no significant differences between study participants who learned through practice compared to participants who learned through our curriculum, our study reveals insights into factors contributing to end-user robot programmers’ confidence and success during programming and how learning interventions may contribute to such factors. Our work paves the way for further research on how to best structure training interventions for end-user robot programmers.

## Introduction

End-users increasingly need to customize the behavior of collaborative robots (cobots), a need which has initiated the development of *end-user robot programming* methods. *Kinesthetic teaching* is one such programming method, allowing users to manually guide a robot instead of writing code to specify task behaviors. Kinesthetic teaching has various advantages, such as making programming robot manipulation accessible to anyone capable of moving a robot, avoiding the correspondence problem by operating directly in the robot’s configuration space, and requiring low cognitive load from users [1].

Despite its advantanges, kinesthetic teaching involves difficulties that hinder end-users from creating effective demonstrations. Users experience challenges programming smooth, collision-free kinesthetic demonstrations because of unfamiliarity with the robot’s motion capabilities and programming interfaces such as teach pendants, as well as the physical demands involved in maneuvering a robot by hand [1, 2]. Effective kinesthetic teaching requires users to develop physical and cognitive skills, such as reasoning capabilities on how to best move a robot to avoid joint limits or obstacles, to overcome these difficulties. Consequently, novice users without such skills are more likely to produce suboptimal demonstrations [3–5].

Suboptimal kinesthetic teaching has primarily been addressed using robot learning algorithms that are robust to suboptimal inputs (*e.g*., [6]), online assistance to help users avoid suboptimalities during kinesthetic teaching (*e.g*., [7]), and interfaces to help users improve their demonstrations (*e.g*., [8]). These approaches minimize the need for the user to produce high-quality demonstrations but fail to address an underlying cause of suboptimal kinesthetic demonstrations—a skills gap preventing users from determining best practices for teaching robots tasks kinesthetically. Therefore, recent work has begun shifting focus toward designing educational tools to help users learn kinesthetic teaching to help address this gap.

### Educational tools for end-user robot programming

Given that a growing share of end-users are working closely with robots, there is increasing interest in developing educational tools to help users develop robot programming knowledge and skills so that they can customize robot behaviors according to their needs and utilize robot capabilities [9]. Educational interventions can not only help with skill development but may also improve users’ perceptions of robots [10], which suggests that developing appropriate educational tools for robot programming may affect not only how effectively users are able to work with robots but also their perceptions and experiences in doing so.

Learning robot programming has traditionally required years of training on topics such as controls, sensing, and text-based coding. With the emergence of cobots, end-users can learn to program over the course of weeks instead of years. Users can learn *task-level* programming through classes on how to deploy, set up, and program robots (*e.g.,* Universal Robots’ UR Academy) or by operating and programming cobots through simulated environments (*e.g*., [11]), telelabs (*e.g*., [12, 13]), or training interfaces (*e.g*., [14]). Users with minimal training may also learn how to abstract programs into sub-tasks for subsequent robot task learning (*e.g*., [15, 16]) in a shorter time frame.

In some scenarios, such as lab-based user research, learning involves a shorter time span of minutes or days. In these scenarios, robot programming education primarily focuses on *interface-level* training for robot programming. Common tools for short-term education include expert demonstrations (*e.g*., [15–17]), practice tasks (e.g., [18]), video tutorials (*e.g*., [19]), and reference sheets or manuals (*e.g*., [19]).

Compared to task-level and interface-level robot programming education, *motion-level* education for kinesthetic teaching has received less attention, and users have conventionally relied on practice and trial and error to develop physical familiarity and intuition on how to manually demonstrate tasks for cobots. However, unstructured practice may not be the best approach in preparing users to kinesthetically teach robots, as users may fail to fully understand the robot’s motion capabilities, particularly when it is not humanlike or when the task at hand is complex [9]. Furthermore, leaving end-users to learn independently may minimize opportunities to anchor their perceptions of a robot’s abilities [20, 21] and to enhance their ability to identify critical features for motor tasks [22].

Prior work has suggested that training interventions may accelerate the development of skills necessary for kinesthetic teaching compared to open-ended practice (*e.g*., [23, 24]). Existing work on education for kinesthetic teaching has focused on understanding the effectiveness of different modalities (*i.e.,* visual or haptic) for training or understanding how to prime end-users to develop kinesthetic demonstrations that are effective for subsequent task-level robot learning (*e.g*., [15, 16]). In this work, we seek to further understand how we can support end-users in motion-level programming. In particular, we investigate how *curriculum-based* training can be used to help end-users learn to kinesthetically teach robots.

### Curriculum-based training

Curricula are planned sequences of instructional activities that are commonly used for skill learning in educational and therapeutic applications. Curricula add structure to learning processes and involve strategic sequencing of learning tasks. Humans acquire a variety of motor skills (*i.e.,* grasping) through curriculum-based learning, where a motion learning problem is broken into a series of smaller learning goals or modules, often sequenced in order of increasing difficulty (*e.g*., [25]). Prior work has suggested that varying task difficulty during learning in a curriculum-based approach can help maximize learning gains in motor skill learning (*e.g*., [26]), and curricula are frequently used for this purpose in physical education, sports, and surgical training applications.

**Fig 1:**
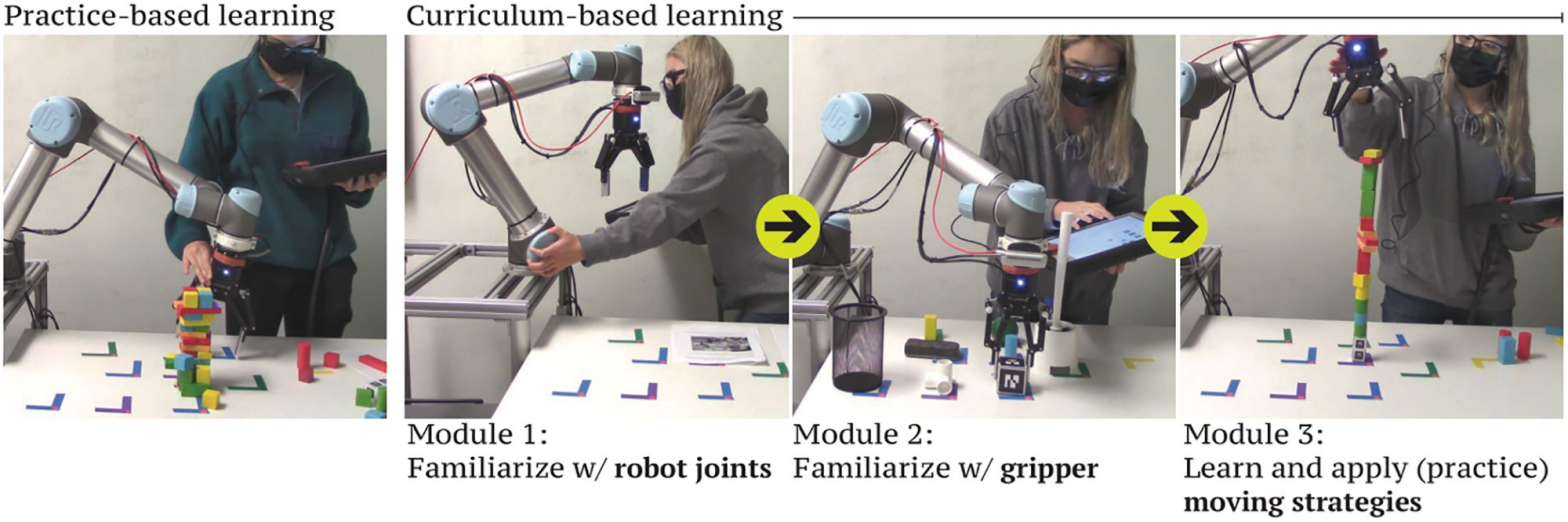
Study conditions. We explore how a curriculum-based approach compares to self-guided practice in helping users learn kinesthetic teaching.

While prior work on curriculum-based learning for robotics has primarily been used to teach robots rather than humans (*e.g*., [27, 28]), recent years have seen the application of curriculum-based learning to teach *humans* how to better use robots, particularly in the areas of robot-assisted surgery (*e.g*., [29, 30]) and rehabilitation (*e.g*., [31]). In this work, we apply curriculum-based learning to kinesthetic teaching and investigate how it impacts users’ programming perceptions and proficiency compared to practice-based learning representative of current training practices for kinesthetic teaching (Fig 1). Our work aims to discover the implications of structured training methods for end-user robot programming. The key contribution of this work is an empirical exploration of the effects of different learning methods on various measures of programming proficiency.

## Materials and methods

We conducted a user evaluation to understand how practice- and curriculum-based learning differ in their effects on programming proficiency and user perceptions. Our study consisted of two one-hour sessions where participants worked on kinesthetic teaching tasks with a UR5 robot arm and teach pendant.

### Study design and experimental tasks

Our two-session study was a between-subjects study, where the first of the two sessions was a learning session with different tasks depending on which of two study conditions the participant was randomly assigned to (Fig 1):

- *Practice*. This condition represents learning by practice. Participants spent the full session programming the UR5 to build a tower as tall as possible using toy blocks, which is similar to the pick-and-place practice tasks users conventionally undergo when learning kinesthetic teaching for lab-based user research. This task involves practicing skills fundamental to kinesthetic teaching using the UR5 (*i.e.,* guiding the robot, grasping objects).
- *Curriculum*. This condition represents curriculum-based learning. Participants completed a curriculum that we designed, described in the next section.

Both learning interventions (practice and curriculum) involved the same amount of time.

**Fig 2:**
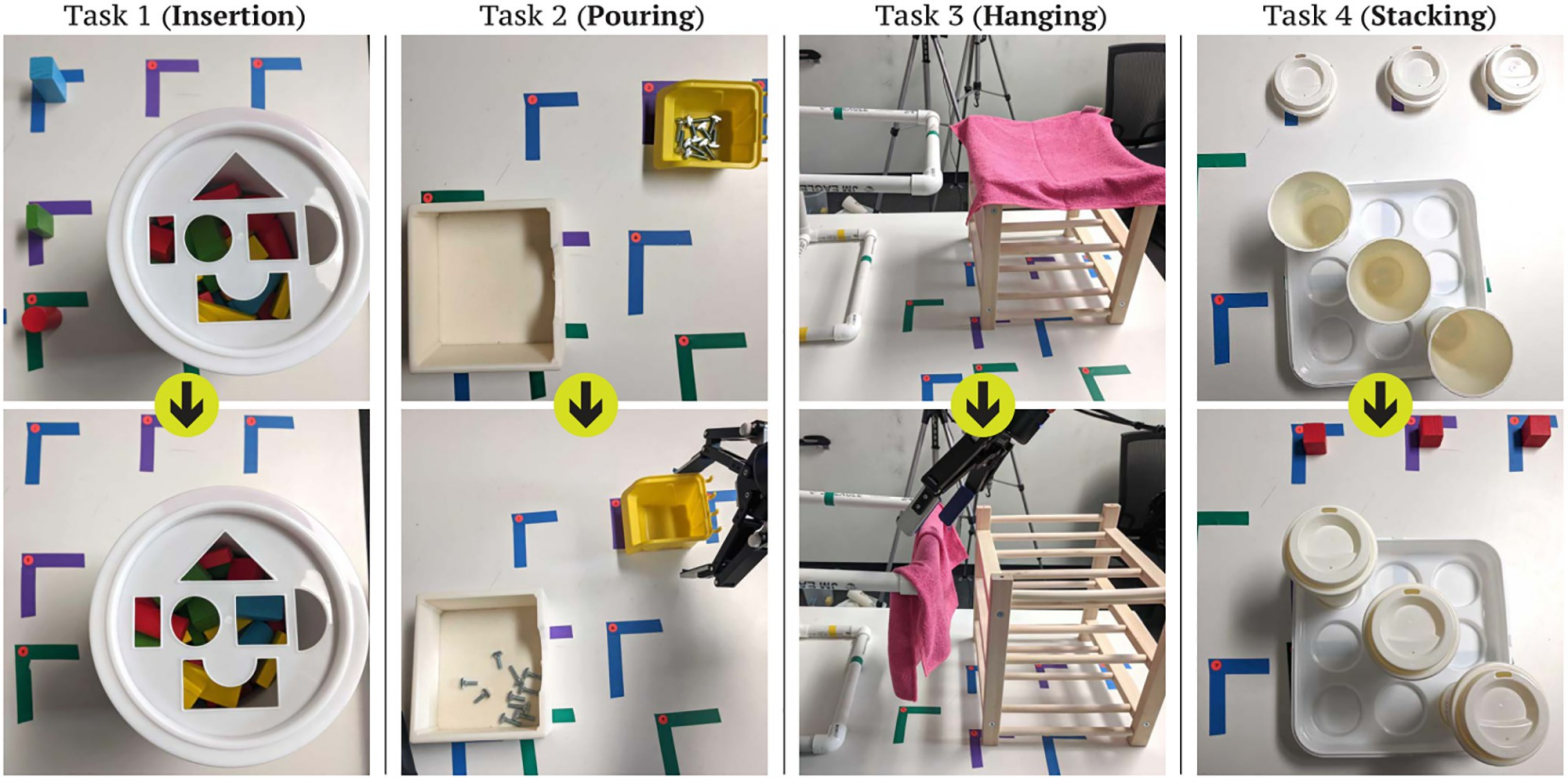
Study tasks testing learned kinesthetic programming skills. Participants completed four tasks that tested their learned skills: Task 1 (insertion), Task 2 (pouring), Task 3 (hanging), and Task 4 (stacking).

The second session was the same across conditions. Participants completed four target tasks testing their learning (Fig 2):

- *Task 1 (insertion)*. Program the robot to pick three differently-shaped blocks and place them into a toy box by inserting them into shaped slots on its lid.
- *Task 2 (pouring)*. Program the robot to pour screws into a container without spilling any.
- *Task 3 (hanging)*. Program the robot to pick up a towel from a shelf and hang it on a makeshift towel rack.
- *Task 4 (stacking)*. Program the robot to stack lids onto coffee cups.

The primary goal of educational interventions is to enable trainees to apply the skills they learn beyond the learning context [32]. Therefore, the four tasks represent actions collaborative robots commonly perform contextualized in real-world application scenarios and included examples of both *near transfer* and *far transfer* [32]. The first and fourth tasks were near transfer tasks that tested whether participants could *replicate* the pick-and-place skills they practiced in the learning session in different contexts, while the second and third tasks were far transfer tasks that tested how well participants were able to *generalize* the skills to manipulation tasks beyond standard pick-and-place. Participants did not practice these tasks directly in the first session for either of the two study conditions. Participants had to press a button on the back of the teach pendant to activate the kinesthetic teaching capabilities of the UR5 and use on-screen buttons to operate the robot’s gripper but otherwise did not have to interact with the teach pendant during kinesthetic teaching.

### Curriculum design

**Fig 3:**
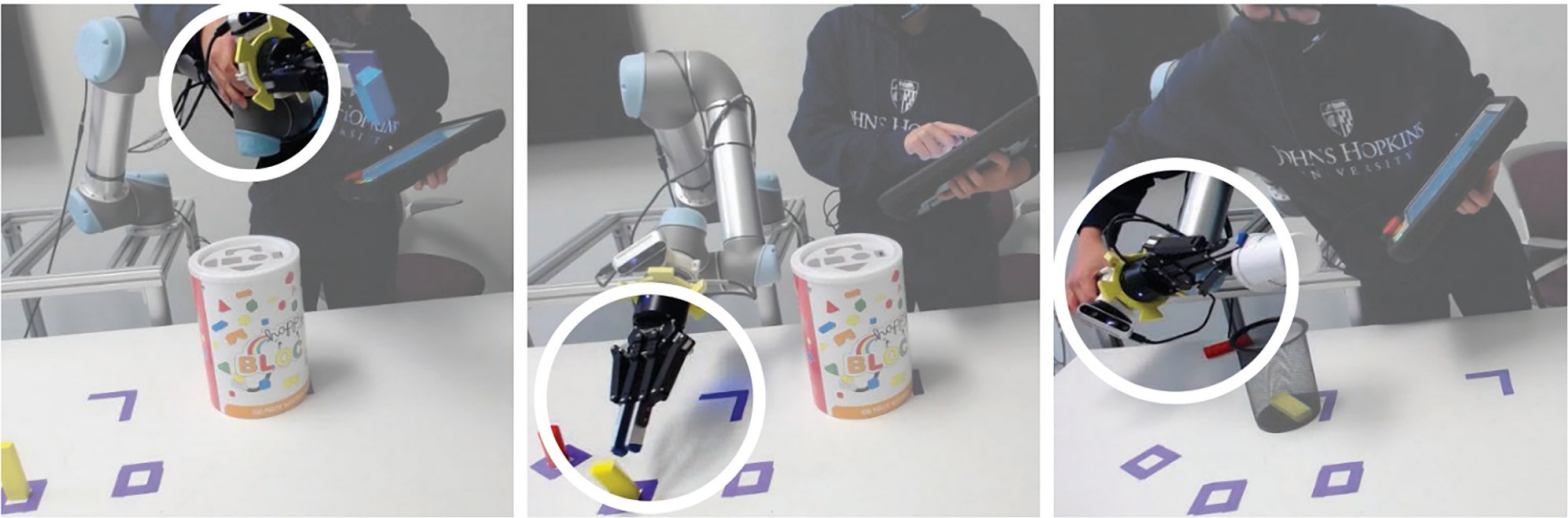
Component skills we identified as necessary for effective kinesthetic teaching. We observed component skills that end-users with low programming task performance lacked: (left) moving individual robot joints, leading to overreliance on the end effector; (middle) gripping; and (right) planning, resulting in errors such as object drops (right).

We developed a curriculum based on findings from learning science and our empirical exploration (S1 File). To identify what critical component skills end-users require to develop good kinesthetic demonstrations, we conducted a pilot study where users performed kinesthetic teaching with a UR5 arm to program various manipulation tasks, with a focus on end-users who had poor task performance because low performance can indicate a lack or weakness in critical component skills [33]. We identified three component skills necessary to effectively program the UR5 kinesthetically: (1) moving individual robot joints, (2) gripping, and (3) planning (Fig 3).

We developed three separate curriculum modules to help users practice each of the component skills. To master an activity, learners must first acquire component skills pertaining to that activity and then integrate and apply them [32]. Furthermore, learners often develop skills more effectively if component skills are practiced in isolation, which can help reduce initial cognitive load while developing fluency [34], and then combined [35–37], and there are advantages to both practicing component skills in isolation as well as in the context of a task [32]. Therefore, we designed the curriculum to follow a bottom-up learning approach consisting of three modules, where end-users first learn the component skills and then learn to integrate and apply the skills in a task. In addition to focusing on component skills, our curriculum incorporated contrast as a teaching strategy and included patterns of variation to help users understand how joint motion relates to end effector positions [22].

Hands-on activities, which are fundamental in motion-level skill learning [12], were incorporated in each of the curriculum modules. Because deliberate practice involving concrete, measurable goals can be more effective than generic, open-ended practice [32], each of the curriculum modules involved specific objectives. The curriculum was proficiency-based: rather than allocating a fixed amount of time or number of repetitions to each module, the learner spent as much time as they needed on each module until they could succeeded in performing the skill being taught. Success was measured by correct placement of target objects in or at the target goal locations. Our curriculum consisted of the following modules (Fig 1, right):

- *Module 1 (component skill: individual joint motion)*. This module aims to familiarize learners with joint rotation and limits. The learner is given a checklist with 12 tasks to complete, where each task involves moving one of the robot’s six joints until it reaches a joint limit in either direction. The learners use the UR5’s teach pendant interface to view the current joint angles as they complete this module. This module involves learners varying a single joint angle at a time while keeping all other joint angles invariant, with the goal of enabling them to understand how joint angle functions as a dimension of variation in specifying end effector positions.
- *Module 2 (component skill: gripping)*. This module aims to familiarize learners with using the robot’s gripper to manipulate objects. The learner is given a checklist with nine tasks to complete, where each task involves using the gripper to grasp an object. Learners could grasp objects however they wanted as long as the teach pendant detected an object in the gripper after their grasp. The objects in this module included everyday objects, such as a PVC pipe, toilet brush, and dry-erase marker. This module allows learners to vary all six joint angles at a time, with the goal of enabling them to learn how to generalize joint angles in specifying different end effector positions.
- *Module 3 (part 1: planning, part 2: component skill integration in task context)*. Novices tend to spend insufficient time planning or plan ineffectively compared to experts [32]. Therefore, this module aims to familiarize learners with planning strategies in moving the robot to demonstrate task behaviors. Learners watch a short video describing strategies on how to smoothly move the robot, choose grasp points to stably manipulate objects, and work with constraints (S1 Video). Then, depending on how much time they have left in the session, they practice applying the strategies to program the UR5 to build a tower as tall as possible using toy blocks (same as the task for the practice condition).

While the curriculum does give learners more exposure to a variety of task objects (particularly in Module 2) compared to open-ended practice, we note that none of the task objects used for the curriculum condition were used in the test tasks in the second study session, other than the blocks used for the tower-building task, which were used for Task 1 and were also used for the learning task in the practice condition. Furthermore, participants in the practice condition have more exposure to the blocks used for Task 1. This design seeked to minimize any advantages in either of the learning interventions in terms of interacting with task objects during kinesthetic teaching.

### Hypothesis and measures

Prior work indicates that training beyond self-guided practice accelerates development of programming proficiency [23, 24], and learning interventions involving patterns of variation can produce more powerful learning than repetition-based practice [22]. Therefore, we hypothesized that *learners following the curriculum we designed will outperform learners following self-guided practice in developing kinesthetic programming confidence and proficiency.* We captured various aspects of perceived and actual programming proficiency by collecting data related to user experience, task success, task efficiency, program quality, and user gaze behaviors (S2 File). We detail the measures we used in this study as well as our hypotheses corresponding to each.

#### User experience

We collected the following data related to participants’ confidence and workload in kinesthetically programming the UR5 arm (S1 Dataset):

- *Change in confidence in UR5 programming*. Custom scale measuring change in confidence in programming using the UR5 between the start and end of the study (Cronbach’s *α* = 0.85); three items assessing the participant’s change in confidence in operating, moving, and programming the UR5. We hypothesized that learners following the curriculum we designed will have a greater positive change in confidence in programming using the UR5 because they were given more direct training on the UR5’s joints and gripping capabilities.
- *Change in confidence in planning and executing kinesthetic teaching*. Custom scale measuring change in confidence in developing kinesthetic demonstrations between the start and end of the study (Cronbach’s *α* = 0.83); three items assessing the participant’s change in confidence in strategically moving the robot and using its gripper without encountering difficulties. We hypothesized that learners following the curriculum we designed will have a greater positive change in confidence in planning and executing kinesthetic teaching because they were given more direct training on strategically moving the robot and gripper during kinesthetic teaching.
- *Projected confidence*. One questionnaire item on a 1-to-5 rating scale about the participant’s projected confidence in programming the robot to complete a task not practiced during the study, transferring dishes from one dish rack to another (task pictured in the questionnaire). We hypothesized that learners following the curriculum we designed will have greater projected confidence in completing an unfamiliar task because they were explicitly trained on task-independent aspects of programming such as how to use and move the robot and how to strategically perform kinesthetic teaching across different task constraints.
- *Adapted NASA TLX*. Modified version of the NASA TLX (Cronbach’s *α* = 0.70); three items measuring users’ workload and effort in programming, which we chose after running exploratory factor analysis on the items we adopted from the original NASA TLX questionnaire. We expected that participants who followed the curriculum may have higher mental demand and effort because they were explicitly trained to consider cognitive aspects of robot programming such as planning and strategizing but may have lower physical demand and effort because they were provided explicit training on the robot’s joints and gripper and how to move them and therefore may have a better idea of how to move the robot effectively. Therefore, we hypothesized that learners following the curriculum may report comparable workload and effort compared to learners following self-guided practice.

#### Task success

We collected the following data related to participants’ task success in kinesthetically programming the UR5 arm (S2 Dataset):

- *Task progress*. Number of tasks for which participant completed a demonstration that achieved the task goal. We hypothesized that learners following the curriculum would be able to complete more tasks.
- *Number of unsuccessful demonstrations*. For each completed programming task, the number of unsuccessful task demonstrations the participant performed before completing a successful demonstration. We hypothesized that learners following the curriculum would have less unsuccessful demonstrations.

#### Task efficiency

We collected the following data related to participants’ task efficiency in kinesthetically programming the UR5 arm (S3 Dataset):

- *Task time (seconds)*. For each completed (successful) programming task, the interval starting when the participant first moved the robot and ending when they completed the task goal (*i.e.,* the last object was placed). Although there is a possibility that learners who followed the curriculum may spend more time planning their programs because they explicitly learned about programming strategies, we hypothesized that task time would be lower among curriculum-based learners compared to practice-based learners because we expected curriculum-based learners to move the robot more effectively and therefore spend less time on corrective motions while programming. Furthermore, we expected most of the time spent planning would occur before the participant began performing the kinesthetic teaching and would therefore not be contained within the task time.

#### Program quality

We collected the following data related to the quality of participants’ kinesthetic demonstrations using the UR5 arm (S4 Dataset):

- *Number of program suboptimalities*. Erroneous actions in programs that did not lead to immediate task failure. We counted the following suboptimalities: gripper collisions with environment; missed grasps; object drops; and robot self-collisions. Our hypothesis was that learners following the curriculum would have less suboptimalities in their programs compared to learners following open-ended practice.
- *Average rates of change of force and torque (Newtons/sec, Newton-meters/sec)*. For each completed programming task, average rates of change of the forces and torques users exerted for successful demonstrations, with higher values indicating jerkier demonstrations. Minimum-jerk trajectories are considered more optimal in robot path planning because they are considered safer for collaborative robots working close with humans [38] and smoother and more natural and therefore more similar to human motions [39]. We hypothesized that curriculum-based learners would produce less jerky demonstrations because they received explicit training on motion strategies to produce smoother motions.

#### User gaze behaviors

We collected the following data related to participants’ gaze behaviors while kinesthetically programming the UR5 arm (S5 Dataset):

- *Gaze fixation duration and quantity*. As prior work has suggested that individuals’ gaze patterns can differ depending on their level of skill in kinesthetic teaching [40] and in other activities involving motor skill such as surgery [41], we collected users’ *fixation durations (seconds)* and *quantities* using a gaze tracker to discover whether fixations differ depending on the type of learning users underwent. In particular, we collected data on fixations on the robot gripper because prior work has indicated that novice users may fixate on the gripper longer than more expert users [40]. We hypothesized that this would translate into our robot programming domain and that curriculum-based learners would gain more expertise in robot programming and would therefore have shorter and less fixations on the robot’s end effector relative to practice-based learners.

We include the covariate data we collecting, excluding protected characteristics, in S6 Dataset.

### Study procedure

The study protocol was approved by the Johns Hopkins University Homewood Institutional Review Board #HIRB00007756 on June 21, 2021. The study procedure consisted of two sessions spaced approximately 24 hours apart:

#### Session 1

After obtaining written informed consent, the experimenter provided the participant with a Pupil Invisible gaze tracker to wear and had them complete a calibration task to verify correct gaze detection. Next, the experimenter provided the participant with a written tutorial on how to use the teach pendant to perform kinesthetic teaching using the UR5, which covered how to physically guide the robot by compressing a button at the back of the teach pendant, open and close the robot’s gripper using on-screen buttons, and use the robot’s safety features as needed. For this study, the participant only needed to use the teach pendant for the purposes of activating its kinesthetic teaching functionality, opening and closing the robot’s gripper, and using the emergency stop button if needed. Therefore, the kinesthetic teaching process did not involve high amounts of on-screen interaction with the teach pendant.

Once the participant finished reading the tutorial, the experimenter answered questions and then instructed them to complete a familiarization task where they kinesthetically program the robot to pick and place a block. Upon successful completion of the familiarization task, the participant completed a pre-study questionnaire. They were then instructed to begin the learning tasks corresponding to their study condition. For both conditions, participants had 40 minutes to complete their respective learning session tasks. A sheet with the practice task instructions or a printed curriculum booklet was provided to the participant depending on the study condition. The experimenter was not present during the tasks except to set up the robot and task objects, unless the participant explicitly requested assistance.

#### Session 2

For the second session, the experimenter provided the participant with the gaze tracker and then instructed them to start the four tasks testing their learned skills. For each task, the experimenter provided a sheet with task instructions to the participant. The experimenter was not present during the tasks except to set up the robot and necessary objects between tasks, unless the participant explicitly requested assistance. The participant had up to 50 minutes to complete the four tasks. Once the participant finished all the tasks or 50 minutes had elapsed, they completed a post-study questionnaire. Finally, the experimenter conducted a short open-ended interview to learn more about the participant’s experience with programming the target tasks and how the learning session contributed to it.

The study was approximately two hours long. Participants were reimbursed 15 USD for each one-hour session they completed.

### Participants

We recruited 28 participants in January 2022; one participant from the practice condition did not complete the second study session and is not included in our analysis. The remaining 27 participants (17 females, 9 males, 1 non-binary) were aged between 18 and 24 (*M* = 20.44, *SD* = 1.89) and reported being slightly experienced with robots (*M* = 2.41, *SD* = 1.12), experienced with technology (*M* = 4.15, *SD* = 0.82), experienced with programming (*M* = 3.96, *SD* = 0.94), and slightly experienced with programming robots (*M* = 2.30, *SD* = 1.27) on 1-to-5 rating scales (1: “No experience,” 5: “Lots of experience”). Participants were students from different backgrounds including engineering, public health, and recording arts. Many of the participants came from Computer Science backgrounds. While some participants had prior experience with robotics, none of the participants were experienced with kinesthetic teaching. 14 of the participants were assigned to the curriculum condition, and 13 were assigned to the practice condition. Among the participants in the curriculum condition, all of the participants started all three modules, but one participant ran out of time after finishing the first part of Module 3 (watching the video on programming strategies) and was therefore unable to do the second part of the module (building the tower).

We excluded some participants from the data analysis for individual task measures due to failure to follow instructions (*i.e.,* manipulating objects directly with their hands, demonstrating the wrong actions), technical failure of the robot or gaze tracker during the study, or failure to complete the task. Due to data loss, some participant data was also excluded from the analysis of average force and torque change. The full list of excluded data is provided in S2 Appendix. The authors did not have access to information that could identify individual participants during or after data collection.

## Results

We describe the results from our analysis on how the type of learning users underwent (*practice, curriculum*) affected our study measures. We modeled each of our measures as dependent variables using statistical models. To determine whether to use a parametric model, we performed tests for normality and homoscedasticity. Details about the models we employed are provided in S3 Appendix. We used the JMP version 16 software to calculate scale reliability for our subjective measures and IBM SPSS version 28 for all other statistical analysis. For qualitative results, we use *#P* to indicate the number of participants in the practice condition and *#C* to indicate the number of participants in the curriculum condition attributed to the statement.

### User experience

**Fig 4:**
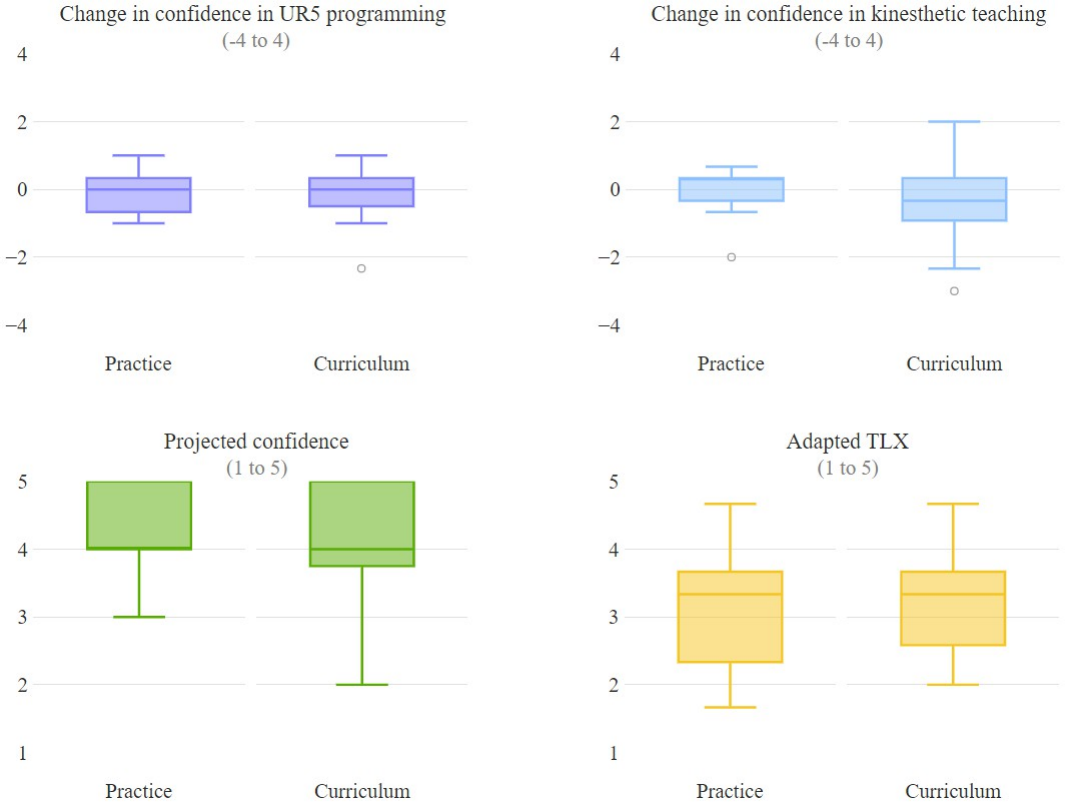
Results on measures of user experience. The top and bottom of each box represent the first and third quartiles, and the line inside each box is the statistical median of the data. The length of the box is defined as the interquartile range (IQR). The ends of the whiskers are the first quartile minus 1.5 IQR and the third quartile plus 1.5 IQR. Circles indicate outliers. A higher value indicates better user experience. We found no significant differences between participants in the two study conditions on measures of user experience.

We found no significant effect of learning method on users’ change in confidence in UR5 programming (*p* = .960), change in confidence in planning and executing kinesthetic teaching (*p* = .357), projected confidence (*p* = .301), and workload and effort (*p* = .083) (Fig 4). Our hypotheses that curriculum-based learners’ would have a greater change in confidence and projected confidence were not supported. Participants who indicated a decrease in confidence in their questionnaires indicated during the interview that their decrease in confidence was due to experiencing more programming failures (*3P, 2C*) and because of the initial familiarization task that they completed just before indicating their initial confidence being too simple, which caused them to be initially overconfident (*2P, 2C*). Participants who indicated an increase in confidence attributed a variety of reasons to their increase in confidence, which included gaining more experience working with different task constraints, getting more practice, having a better understanding of the robot toward the end of the study, overcoming the learning curve, and succeeding during the target tasks. Participants described similar factors, primarily related to increasing levels of experience and understanding, as affecting their confidence across both study conditions.

**Fig 5:**
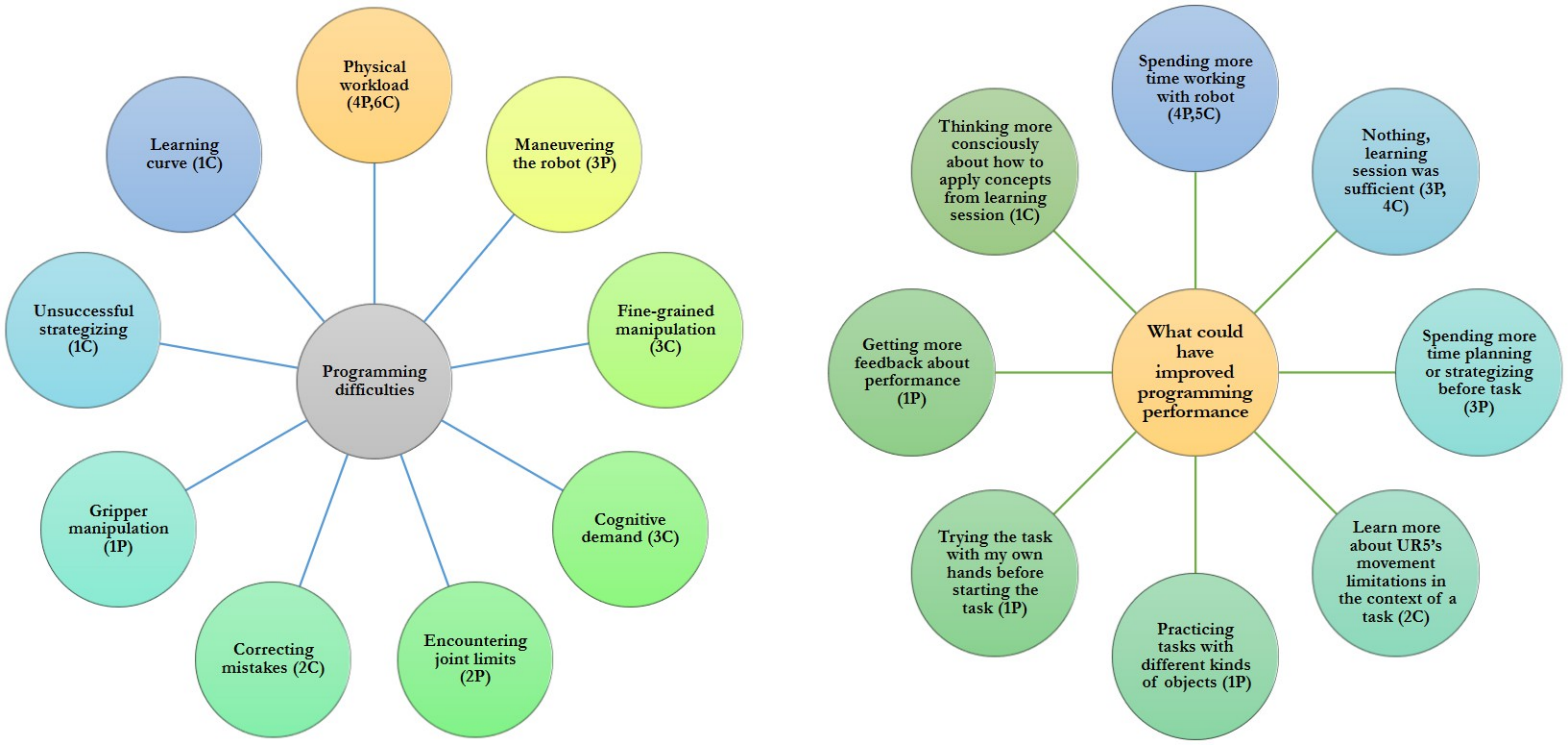
Participants’ programming challenges and preferences for additional preparation. We summarize participants’ comments about what they found challenging during programming and what could have prepared them to face programming challenges beyond the learning session they previously completed.

Most participants across both conditions indicated that the most challenging part of programming was the physical demand involved in using the programming system (*4P,6C*) (Fig 5, left). Participants in the curriculum condition mentioned fine-grained manipulation (*3C*) and the cognitive demand involved in programming (*3C*) as other challenges they faced, which supported our hypothesis that participants may have similar overall workload across conditions but that curriculum-based learners may have higher cognitive workload related to strategizing and considering task constraints in tasks involving fine-grained gripper movements. Participants in the practice condition described challenges related to maneuvering the robot (*3P*) and encountering joint limits (*2P*) more than curriculum-based learners, which partially supported our hypothesis that practice-based learners may have higher workload related to moving the robot.

### Task success

Our analysis yielded no significant differences between participants in our two study conditions in terms of task success. Several participants indicated that the target tasks in the second session were easier than the learning tasks in the first session (*4P, 4C*). However, two participants in the practice condition indicated that they found the target tasks harder than the learning tasks. Several participants indicated that they found Task 2 (pouring) (*2P,1C*) or Task 4 (stacking) (*3P,1C*) the most difficult among the target tasks.

#### Task progress

In the practice condition, all participants completed all four of the target tasks, while one of the participants in the curriculum condition did not complete the fourth target task. There was no statistically significant difference in task progress between the two study conditions (*p* = .254). Our hypothesis that curriculum-based learners would complete more tasks was not supported.

#### Number of unsuccessful demonstrations

There was no significant difference in number of unsuccessful demonstrations between participants in the two study conditions (*p* = .516). Our hypothesis that curriculum-based learners would have less unsuccessful demonstrations was not supported.

Participants who experienced success (perceived or actual) during programming attributed their success to intuition (*2P*), planning or forethought (*2P, 1C*), or application of knowledge they gained from previous tasks (*3C*).

### Task efficiency

There was no significant difference in task times between participants in the two study conditions (*p* = .727). Our hypothesis that curriculum-based learners would spend less time to complete programming tasks was unsupported.

### Program quality

Participants in our two study conditions had a similar amount of suboptimalities and jerkiness in their demonstrated motions.

#### Number of program suboptimalities

There was no significant difference in the number of suboptimalities in participants’ demonstrations between the two study conditions (*p* = .125). Our hypothesis that curriculum-based learners would have less suboptimalities in their programs was unsupported.

#### Average rates of change of force and torque

There was no significant difference in changes in force (*p* = .711) and torque (*p* = .732) between participants’ demonstrations in the two study conditions. Our hypothesis that curriculum-based learners would have less jerky programs was unsupported.

### User gaze behaviors

Participants in the curriculum condition had similar amounts and durations of fixations across the two conditions. In particular, there was no significant different in the count (*p* = .508) or durations (*p* = .451) of fixations participants made on the robot gripper between the two study conditions. Therefore, our hypothesis that curriculum-based learners would have less and shorter fixations on the robot’s gripper during kinesthetic teaching due to increased expertise was unsupported.

### Additional qualitative findings

We describe our key findings from interviews with users on their perceptions of learning kinesthetic teaching.

**Fig 6:**
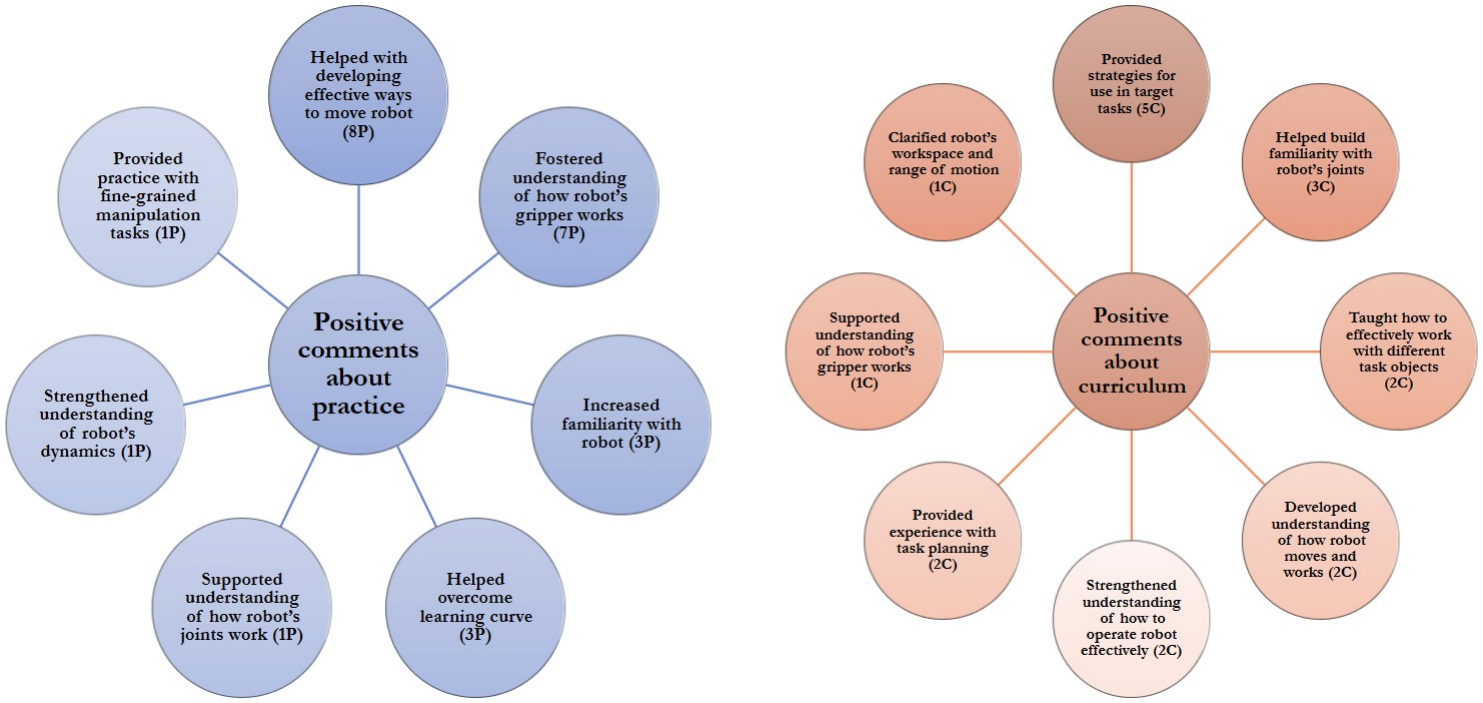
Participants’ positive feedback about the learning session. We summarize participants’ positive comments about the practice or curriculum they completed during the learning session.

*Users’ perceptions of the learning session*. Overall, participants viewed both the practice task and the curriculum as helpful for learning (Fig 6). Participants in the practice condition viewed learning by practice favorably. Compared to curriculum-based learners, practice-based learners more often mentioned that the learning intervention helped them develop understanding of the robot’s gripper (*7P, 1C*), which may be attributed to the increased focus of the practice task on manipulation compared to the curriculum.

Some participants mentioned that the key factors limiting the usefulness of the Session 1 practice was that it did not require the user to get familiar with the robot’s workspace limits, since users could choose to build the tower in the practice task far from the robot’s limits (compared to Session 2, where task locations were predetermined and could be closer to the robot’s limits), and did not include a diversity of objects. Some participants also indicated that they would want more explicit instruction about the robot’s joints and motion (*2P*).

Participants in the curriculum condition generally favored the curriculum modules equally (Module 1: *6C*, Module 2: *4C*, Module 3: *5C*, equal: *1C*; some participants indicated they favored two modules equally and are counted toward both modules). One participant mentioned that the second module was less helpful because *“it was just managing whether or not we actually pick [the objects] up. But then you know, it’s very difficult, different, from like actually picking up a object that is much closer to the surface”* (P13). Some participants liked all of the modules equally, with one participant referring to the necessity of each module to the bottom-up learning approach: *“I think they’re about equal because I can see the progression of like, first getting used to the general movements and the like, gripping it and the like, trying to move stuff around with it”* (P12). Several participants indicated that they directly applied strategies they learned from the curriculum to complete the target tasks (*5C*); however, one participant stated that they had forgotten most of the curriculum content by the second session and therefore did not think to apply what they learned to the target tasks.

*Experienced users’ perceptions of curriculum-based learning*. Among participants with previous experience with the UR5, participants mentioned that the curriculum was still able to help them with kinesthetic teaching despite their familiarity with the UR5. One participant said, *“I would [initally] say the curriculum wasn’t helpful to me because most of what was in the curriculum I already knew but, given that, the curriculum had gave me practice for picking up the blocks and getting the gripper to align with them perfectly and essentially gave me the idea that I should be controlling the robot…joints directly instead of trying to just pull on the gripper”* (P23). Another participant mentioned that although they had worked with the UR5 as part of a robotics course, programming the UR5 by coding did not prepare them for kinesthetic teaching, and the curriculum helped them gain intuition on how to manually move the robot.

*Users’ perceptions of the role of practice in determining task confidence and success*. Participants described fine-grained alignment motions and limitations of the programming system and robot (see Discussion) as the most difficult aspects of the target tasks, with fewer participants mentioning higher-level strategizing as challenging (Fig 5, left). Although several participants stated the importance of pre-planning and forethought in achieving high task performance, most participants in both conditions said that it was interacting with the robot over time and determining actions based on trial and error that were most helpful to them in developing good demonstrations (Fig 5, right). Similarly, participants mentioned that the amount of practice they had with the robot was the key contributor in building their programming confidence. Based on our interviews, learning by practice and trial and error appears to be paramount in forming positive perceptions of task performance and confidence in users.

## Discussion

In this work, we evaluated the effectiveness of open-ended practice and curricula in teaching end-users to perform kinesthetic teaching. Our goal was to determine whether adding structure to users’ initial learning through curricula could increase users’ programming proficiency compared to conventional unstructured, practice-based learning.

Overall, both practice-based learners and curriculum-based learners had similar performance across all of our study measures, indicating that the practice task and curriculum we compared in this study had a comparable effect on participants’ programming performance. Therefore, our hypothesis that curriculum-based learners would outperform practice-based learners in developing programming confidence and proficiency was unsupported overall. However, our interview data provided insight into participants’ perceptions and experiences that may have contributed to the similarity in outcomes between practice-based learners and curriculum-based learners, which drives our future work and highlights limitations in our study.

### Future work and limitations

While our study findings did not indicate major differences between the types of learning we aimed to compare, our work opens up future directions for investigation on how users develop programming proficiency and possibilities in curriculum-based learning for end-user robot programming. Our study highlighted that participants may focus on different aspects of a training intervention. For example, some participants described the open-ended practice as being primarily focused on practicing fine-grained manipulation, while others mentioned that the open-ended nature of the practice task emphasized the development of task planning skills over manipulation skills. Similarly, some participants described the curriculum as primarily being effective for building fundamental knowledge of the robot’s motion capabilities, while others focused more on its teachings regarding strategies for working with higher-level task constraints. Given the variety of learners’ areas of focus during training, future work could investigate how users’ distribution of learning effort (*i.e.,* how much time they spent on each curriculum module or how much time they spent practicing or strategizing) impacts their proficiency.

Learning, particularly at the motion-level, can involve high individual variation affecting the rate and level to which users develop skills [42], and the ability to develop effective robot programs may be influenced by end-user demographics [15]. Future work on curriculum-based learning should explore whether curricula can help produce more uniform learning outcomes that can surpass performance variability due to individual differences in initial skill. In this study, participants were young adults and largely came from similar educational backgrounds and had comparable amounts of technical experience. In particular, our study participants primarily came from technical backgrounds such as Computer Science. These participants, although having no prior experience with kinesthetic teaching specifically, may be at a more advanced learning level than completely non-technical users and therefore may have found our component skill-based learning approach less useful than novice learners would have [34, 43].

Furthermore, their confidence in kinesthetic teaching may be higher than non-technical users, which may have limited the effect of training interventions on our subjective measures of perceived proficiency. Because our participants were largely familiar with programming and technology, additional studies with more diverse users are necessary to understand how non-technical users learn kinesthetic teaching. A future avenue for exploration is whether moving beyond the convention of leaving users to self-guided practice for learning kinesthetic teaching toward structured training conventions such as curricula can help drive kinesthetic teaching toward improved accessibility for a wider range of users at different skill levels.

Our study design included limitations that may have constrained our investigation of curriculum-based learning. Participants used the standard UR5 programming system to perform kinesthetic teaching, which requires them to use a teach pendant with one hand while maneuvering the robot in the other. This may have made programming excessively burdensome regardless of the user’s skill, which was highlighted by the fact that most participants mentioned physical demand as the primary challenge involved in programming the UR5. Further investigation is required on how users’ performance would differ with two-handed demonstrations or more lightweight robots.

Our work focused on the effect of curricula on improving participants’ programs when viewed as individual trajectories without contextualizing them in the context of teaching robots in a learning from demonstration context, where different aspects of programs that we did not capture in this study, such as abstraction [15, 16] and conciseness [16], may be more important. For example, prior work has indicated that end-users may not naturally provide effective abstraction of motion demonstrations (*i.e.,* specification of subtasks) when providing motion demonstrations and that written curricula may not be helpful for learning effective abstraction [15]. Therefore, further work is necessary to understand how curriculum-based learning may apply to different aspects of robot program specification such as abstraction or authoring that goes beyond trajectory-level programming, such as developing task plans [44].

Learning is often a long-term and iterative process, and short-term user studies may provide a limited view of users’ programming performance beyond initial learning [45]. In fact, one of the study participants pointed to the learning curve as being one of the most difficult aspects of programming the robot (Fig 5, left). Extensive work has shown that one or two trials may be insufficient for new knowledge to be retained and applied to different contexts (*e.g*., [32]). Our study included two one-hour sessions on consecutive days, which may not have been sufficient for users to finish learning and determine how to apply their learned skills in new contexts. In fact, one participant directly stated that they had forgotten most of the content of the curriculum by the time they came in for the second session the next day. The short-term nature of the study also constrained users to practicing each curriculum module once, without sufficient opportunities to further refine their skill across multiple repetitions. Therefore, longer-term studies will be necessary to fully observe the evolution of users’ learning beyond initial learning effects.

Our curriculum is only one example of curriculum-based learning, and different curriculum designs may produce more salient improvements in end-users’ programming proficiency. Our curriculum focused on addressing common areas of weakness in users’ component skills. An alternate approach could instead make learning goals more explicit by emphasizing target performance for users to work toward by giving examples of high-quality or expert demonstrations that end-users can try to replicate [15, 16, 24], which may help users in learning what critical aspects indicate optimal kinesthetic teaching performance and understanding expert programmers’ metacognitive processes [32]. Future work should also explore alternate techniques for sequencing curriculum modules besides bottom-up approaches, such as ordering modules by increasing difficulty or by task domain (*e.g*., [16]), and additional studies on the componenent skills required for kinesthetic teaching.

Furthermore, effective learning requires both practice and feedback [32]. Our curriculum-based approach only included minimal feedback for users at the action-level (*i.e.,* indicating how far they are from reaching a joint position or closing in on an object during gripping). One participant in the curriculum condition mentioned that they would have preferred to have more feedback on the quality of their program during the study. In the future, we would like to explore whether higher-level feedback can result in more effective learning and how to best structure and time feedback to foster programming proficiency development. Our curriculum was also uniform across participants. For future work, we would like to investigate whether tailoring learning interventions to individual end-users to provide them with the appropriate difficulty level and content based on, for example, an initial skill assessment or questionnaire about their personality or perception of robots (*e.g*., [16]) can improve learning outcomes.

Structure is only one among many factors that contribute to effective learning. We hope this work will drive further research into how additional factors in teaching users such as scaffolding, personalization, timing, and mode of delivery can contribute to effective physical and cognitive skill development for kinesthetic teaching. Although our work did not highlight major differences between open-ended practice and a curriculum that we designed, it did emphasize that end-users’ initial experiences in learning programming, including early failures and successes, can play a key role in affecting their confidence and perceptions of programming. Furthermore, some practice-based learners indicated a desire for more structured and direct instruction, indicating that, depending on the learner, adding structure to training interventions may be key in improving user experiences and performance. With this initial work, we highlight the need to consider best practices for teaching and training end-users to make programming methods such as kinesthetic teaching truly accessible for non-experts.

## Supporting information

S1 File Curriculum booklet. This file is the curriculum booklet that we provided to participants consisting of three training modules.
(PDF)

S2 File Summary of study data. This file summarizes the study data we collected and provide as a set of six datasets.
(PDF)

S1 Video Module 3 video. This video goes through different strategies for developing task demonstrations and was shown to users at the beginning of the third curriculum module.
(MP4)

S1 Dataset User experience dataset. This spreadsheet contains the data corresponding to the measures related to user experience.
(CSV)

S2 Dataset Task success dataset. This spreadsheet contains the data corresponding to the measures related to task success.
(CSV)

S3 Dataset Task efficiency dataset. This spreadsheet contains the data corresponding to the measure related to task efficiency.
(CSV)

S4 Dataset Program quality dataset. This spreadsheet contains the data corresponding to the measures related to program quality.
(CSV)

S5 Dataset User gaze behaviors dataset. This spreadsheet contains the data corresponding to the measures related to user gaze behaviors.
(CSV)

S6 Dataset Covariate dataset. This spreadsheet contains the data corresponding to the covariates we used in our models, excluding protected characteristics.
(CSV)

S1 Appendix Confidence questionnaire items. This appendix includes the questionnaire items we used to measure participants’ confidence with various aspects of programming.
(PDF)

S2 Appendix Excluded study data. This appendix contains a list of data that was excluded from our analysis.
(PDF)

S3 Appendix Statistical models. This appendix lists the models we used for our statistical analysis.
(PDF)
