# Supplementary material for: Curricula for teaching end-users to kinesthetically program collaborative robots
Source: PLoS One. 2023 Dec 1;18(12):e0294786. doi: 10.1371/journal.pone.0294786 (PMC10691692; doi:10.1371/journal.pone.0294786)
Supplement: S1 File — This file is the curriculum booklet that we provided to participants consisting of three training modules. (PDF) [file pone.0294786.s001.pdf]

Robot Programming Training Curriculum Booklet

<u>Directions</u>: Please complete each of the training modules below in order. You may request help from the experimenter if you have trouble understanding any of the instructions. Please <u>do not</u> write on this booklet.

<u>Module #1: Moving Joints</u>

In this module, you will practice moving the robot using each of its joints.

The robot you are using has six joints (highlighted in Figure 1 below) that can be rotated, which are labeled in Figure 1:

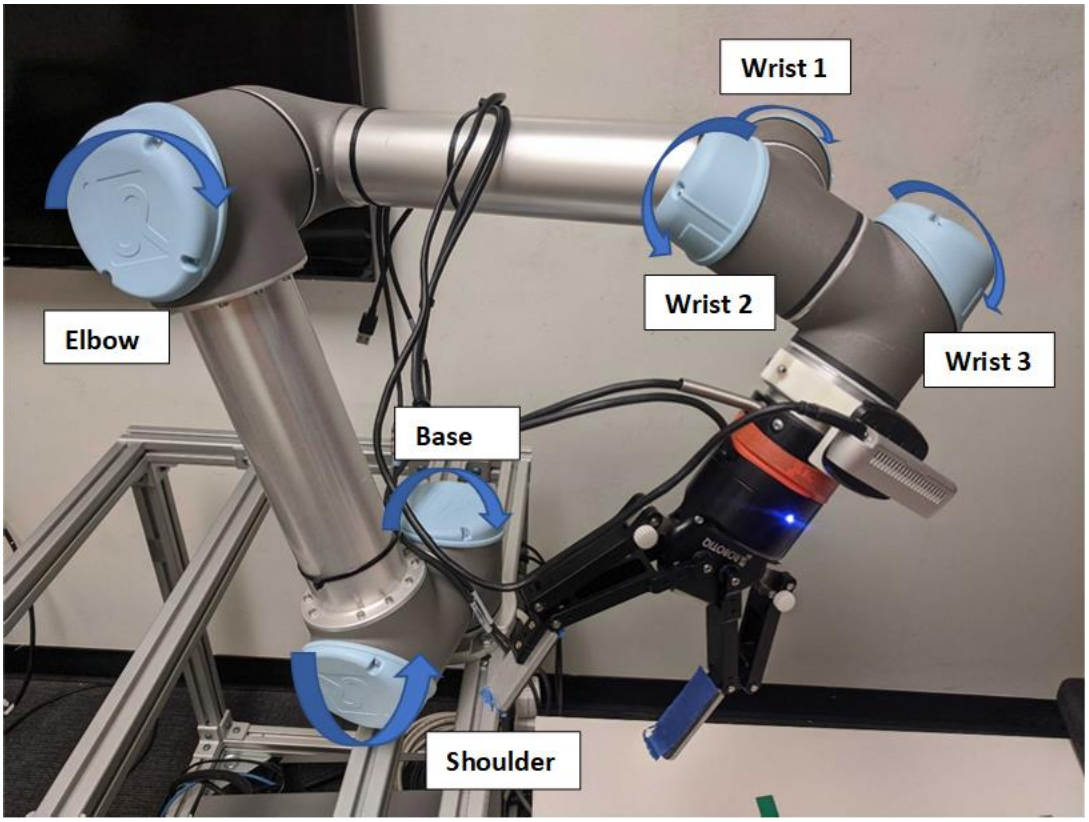

Figure 1: The six joints of the robot, which can be rotated in either direction

The robot's joints can be rotated clockwise and counterclockwise.

You can view the current position of each of the robot's joints, which is presented as an angle between -360° and 360°, on the teach pendant interface in the area outlined in the figure on the next page:

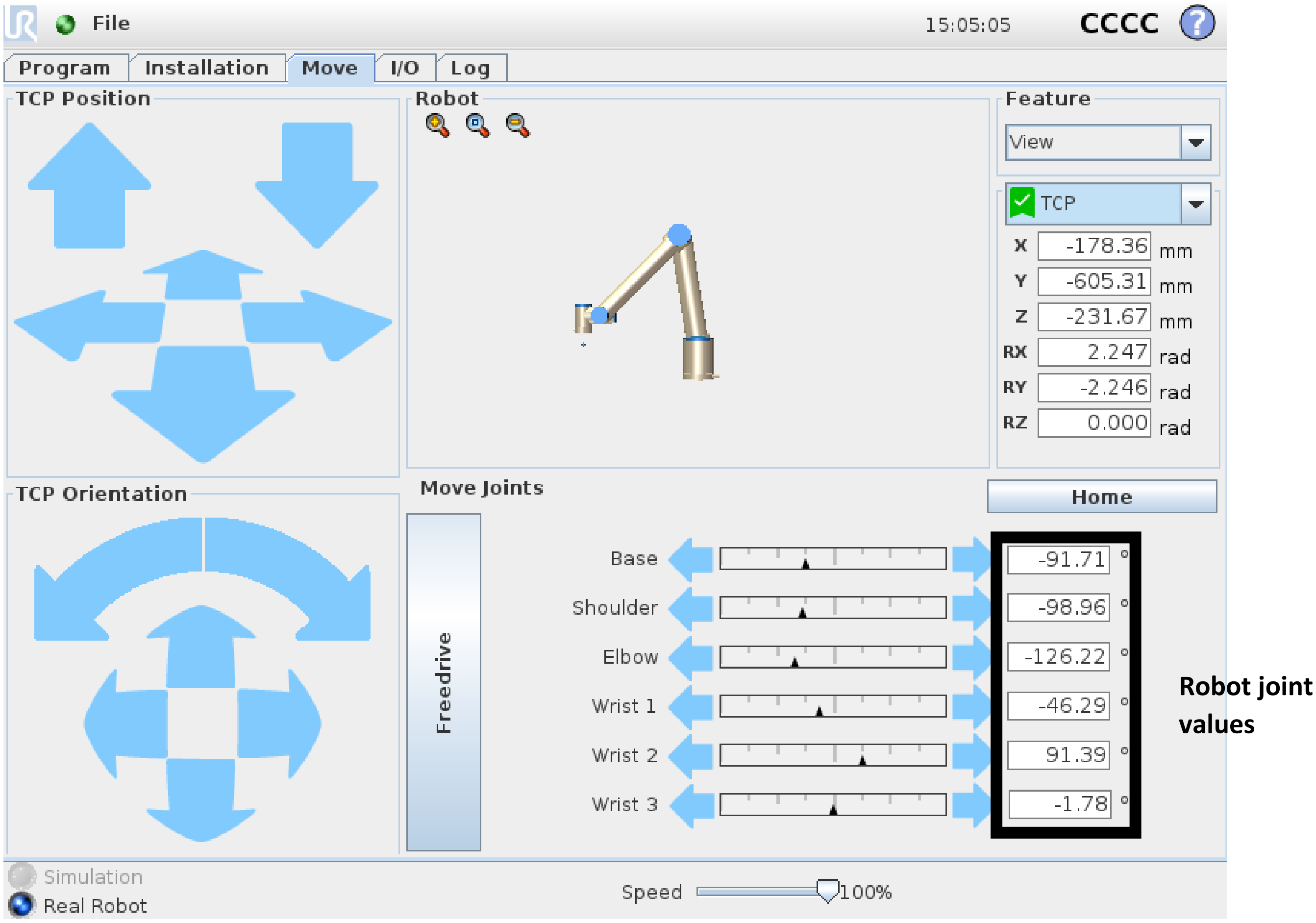

Figure 2: Teach pendant interface, outlined area displays the angular position of each of the robot's joints

For example, we can see that the "Elbow" joint of the robot is currently positioned at -126.22°.

You will now move the robot through a range of different joint positions by guiding the robot by hand using the Freedrive button *(**do not** use the blue arrows on the interface to move the robot)*, while using the teach pendant interface to monitor the robot's current joint values.

Please complete the tasks in the Module 1 Checklist on the Robot Programming Training Curriculum Checklists sheet. Once you have finished all the tasks in the checklist, please indicate to the experimenter that you have completed the module before you proceed to Module 2.

Module #2: Gripping Objects

In this module, you will practice gripping various objects. See Figure 3 on the next page to see the teach pendant features you will be using for this module.

There are nine objects at taped, number locations in the workplace in front of you. For each task in this module, you will grip one of these objects using the close button on the teach pendant. If the gripper detection indicator states that the object is detected in the grip, you can proceed to the next task. Otherwise, please repeat the task using different grasp configurations until the object being gripped is

detected. Please complete the tasks in the Module 2 Checklist on the Robot Programming Training Curriculum Checklists sheet. **<u>Please indicate to the experimenter that you have completed the module before you proceed to Module 3.</u>**

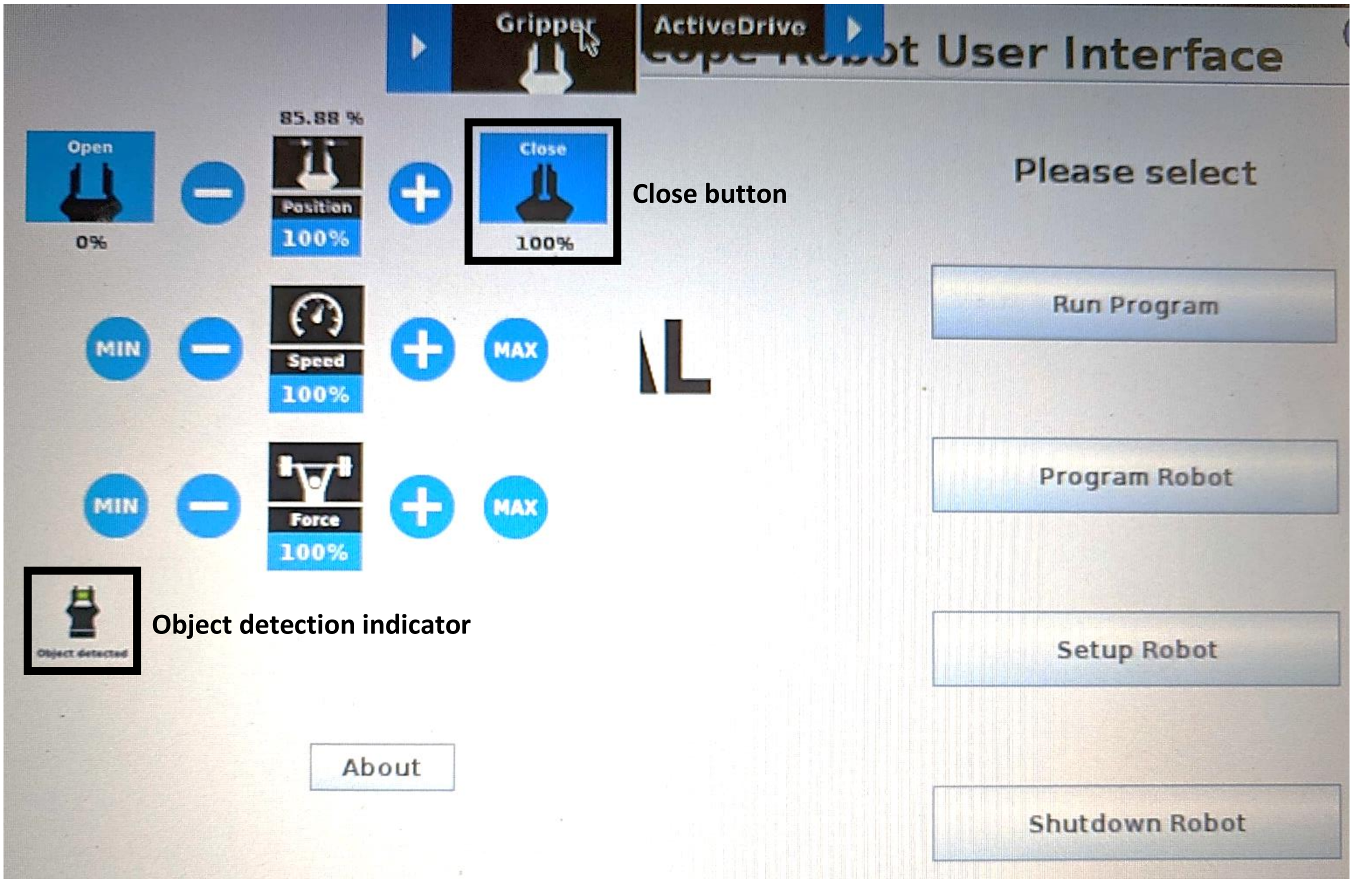

Figure 3. Gripper menu with relevant areas outlined. Use the close button to grip objects. If the object detection indicator states "Object detected", proceed to the next task; otherwise, repeat the task.

<u>Module #3: Programming Strategies</u>

In this module, you will apply what you practiced in Modules 1 and 2 to strategically program the robot to complete a task.

You just finished watching a video describing various strategies programming task demonstrations for the robot. You will now apply those strategies for a programming task.

You have been provided with a container full of blocks. Your task is to program the robot to build a tower using these blocks, with the goal of making the tower **as tall as possible**. You will work on this task for the remainder of the study. During this time, please continue repeating the task until the experimenter indicates that you can stop. Anytime during this task, you can place the blocks on the table for the robot to pick up (i.e., you do not need to program the robot to pick up the blocks from inside the container).

When you are ready to begin the task, please start the recording on the phone app attached to your gaze tracker and then clap once while looking at your hands. When the experimenter tells you to stop

the task, clap once while looking at your hands and then stop the recording on the phone app. You may request help from the experimenter if you have any questions.

Robot Programming Training Curriculum Checklists

Module 1 Checklist

Once you've completed a task, place a checkmark in the box beside it:

**Task**	*Check*
Rotate the ***Base*** joint counterclockwise until it reaches position 180° (approximately)	
Rotate the ***Base*** joint clockwise until it reaches position 80° (approximately)	
Rotate the ***Shoulder*** joint counterclockwise until it reaches position 5° (approximately)	
Rotate the ***Shoulder*** joint clockwise until it reaches position -60° (approximately)	
Rotate the ***Elbow*** joint counterclockwise until it reaches position -45° (approximately)	
Rotate the ***Elbow*** joint clockwise until it reaches position –105° (approximately)	
Rotate the ***Wrist 1*** joint clockwise until it reaches position 80° (approximately)	
Rotate the ***Wrist 1*** joint counterclockwise until it reaches position -105° (approximately)	
Rotate the ***Wrist 2*** joint counterclockwise until it reaches position -90° (approximately)	
Rotate the ***Wrist 2*** joint clockwise until it reaches position 90° (approximately)	
Rotate the ***Wrist 3*** joint counterclockwise until it reaches position -200° (approximately)	
Rotate the ***Wrist 3*** joint clockwise until it reaches position -100° (approximately)	

Flip over ⤷

Module 2 Checklist

Once you've completed a task, place a checkmark in the box beside it.

Please press record on the phone attached to your gaze-tracking glasses and then clap your hands once to indicate that you have started the tasks for this module.

**Task**	*Check*
Grip the object at location **1**. Check if the object is detected in the grip. If not, repeat this task.	
Grip the object at location **2**. Check if the object is detected in the grip. If not, repeat this task.	
Grip the object at location **3**. Check if the object is detected in the grip. If not, repeat this task.	
Grip the object at location **4**. Check if the object is detected in the grip. If not, repeat this task.	
Grip the object at location **5**. Check if the object is detected in the grip. If not, repeat this task.	
Grip the object at location **6**. Check if the object is detected in the grip. If not, repeat this task.	
Grip the object at location **7**. Check if the object is detected in the grip. If not, repeat this task.	
Grip the object at location **8**. Check if the object is detected in the grip. If not, repeat this task.	
Grip the object at location **9**. Check if the object is detected in the grip. If not, repeat this task.	

Once you have finished all the tasks in the checklist above, ***please clap your hands once to indicate that you have finished the tasks and then press stop on the phone attached to your gaze-tracking glasses.***
