# Supplementary material for: Curricula for teaching end-users to kinesthetically program collaborative robots
Source: PLoS One. 2023 Dec 1;18(12):e0294786. doi: 10.1371/journal.pone.0294786 (PMC10691692; doi:10.1371/journal.pone.0294786)
Supplement: S2 File — This file summarizes the study data we collected and provide as a set of six datasets. (PDF) [file pone.0294786.s002.pdf]

Summary of study data

We provide datasets containing the data we used for analysis of each of our study measures. We summarize information about each of the datasets below:

S1_Dataset.csv (Corresponding to *0.3.1 User experience*)

- *UserID*: Participant ID used across all datasets
- *Condition*: Study condition participants participated in
- *ChangeConfUR5Scale*: Data from custom scale measuring change in confidence in programming using the UR5 between the start and end of the study
- *ChangeConfKTScale*: Data from custom scale measuring change in confidence in developing kinesthetic demonstrations between the start and end of the study
- *ProjConf*: Data from questionnaire item about participants' projected confidence in programming the robot to complete a dish transfer task
- *AdaptedTLXScale*: Data from adapted NASA TLX scale
- *PreConfProg, PreConfOp, PreConfMove, NegPreConfErr, PreConfGrip, PreConfDiff, PreConfStrat*: Data from the items listed in pre-study questionnaire (in order shown in *S1 Appendix*); note that the item corresponding to *NegPreConfErr* uses reverse wording
- *PostConfProg, PostConfOp, PostConfMove, NegPostConfErr, PostConfGrip, PostConfDiff, PostConfStrat*: Data from the items listed in post-study questionnaire (in order shown in *S1 Appendix*); note that the item corresponding to *NegPostConfErr* uses reverse wording
- *DiffConfProg, DiffConfOp, DiffConfMove, DiffConfErr, DiffConfGrip, DiffConfDiff, DiffConfStrat*: Difference between data corresponding to participants' post-study confidence items and pre-study confidence items (accounting for reverse wording). *DiffConfProg, DiffConfOp*, and *DiffConfMove* were used to calculate *ChangeConfUR5Scale*. *DiffConfGrip, DiffConfDiff*, and *DiffConfStrat* were used to calculate *ChangeConfKTScale*
- *TLXMental, TLXPhysical, TLXTemporal, TLXPerformance, TLXEffort, TLXFrustration*: Data from the six items in the NASA TLX scale (https://humansystems.arc.nasa.gov/groups/tlx/downloads/TLXScale.pdf) adapted to 5-point Likert scales (1: very low, 5: very high). *TLXMental, TLXPhysical*, and *TLXEffort* were used to calculate *AdaptedTLXScale*

S2_Dataset.csv (Corresponding to *0.3.2 Task success*)

- *UserID*: Participant ID used across all datasets
- *Condition*: Study condition participants participated in
- *TaskProgress*: Number of tasks for which participants completed a demonstration that achieved the task goal
- *T1UnsucDemos, T2UnsucDemos, T3UnsucDemos, T4UnsucDemos*: Number of unsuccessful task demonstrations participants performed before completing a successful demonstration in Tasks 1, 2, 3, and 4, respectively

- *TotalUnsucDemos*: Number of unsuccessful task demonstrations participants performed before completing a successful demonstration, summed across all tasks

S3_Dataset.csv (Corresponding to *0.3.3 Task efficiency*)

- *UserID*: Participant ID used across all datasets
- *Condition*: Study condition participants participated in
- *T1Time, T2Time, T3Time, T4Time*: Time in seconds between when participants first moved the robot and when they completed the task goal for Tasks 1, 2, 3, and 4, respectively
- *TotalTaskTime*: Time in seconds between when participants first moved the robot and when they completed the task goal, summed across all tasks

S4_Dataset.csv (Corresponding to *0.3.4 Program quality*)

- *UserID*: Participant ID used across all datasets
- *Condition*: Study condition participants participated in
- *T1Suboptimalities, T2Suboptimalities, T3Suboptimalities, T4Suboptimalities*: Number of erroneous actions in programs that did not lead to immediate task failure for Tasks 1, 2, 3, and 4, respectively
- *TotalSuboptimalities*: Number of erroneous actions in programs that did not lead to immediate task failure, summed across all tasks
- *AvgAllTasksAvgXForceChange, AvgAllTasksAvgYForceChange, AvgAllTasksAvgZForceChange*: Force participants exerted in the x, y, and z dimensions, respectively, averaged across all tasks.
- *AvgAllTasksAvgXTorqueChange, AvgAllTasksAvgYTorqueChange, AvgAllTasksAvgZTorqueChange*: Torque participants exerted in the x, y, and z dimensions, respectively, averaged across all tasks.
- *T1AvgDimsForceChange, T2AvgDimsForceChange, T3AvgDimsForceChange, T4AvgDimsForceChange*: Force participants exerted averaged across all dimensions for Tasks 1, 2, 3, and 4, respectively
- *T1AvgDimsTorqueChange, T2AvgDimsTorqueChange, T3AvgDimsTorqueChange, T4AvgDimsTorqueChange*: Torque participants exerted averaged across all dimensions for Tasks 1, 2, 3, and 4, respectively
- *AvgAllDimsAllTasksForceChange*: Force participants exerted averaged across all dimensions and tasks
- *AvgAllDimsAllTasksTorqueChange*: Torque participants exerted averaged across all dimensions and tasks

S5_Dataset.csv (Corresponding to *0.3.5 User gaze behaviors*)

- *UserID*: Participant ID used across all datasets
- *Condition*: Study condition participants participated in
- *T1GripperCount, T2GripperCount, T3GripperCount, T4GripperCount*: Number of fixations participants had on the robot's gripper for Tasks 1, 2, 3, and 4, respectively

- *T1BodyCount, T2BodyCount, T3BodyCount, T4BodyCount*: Number of fixations participants had on the robot's body (any part of the robot besides the gripper/wrist) for Tasks 1, 2, 3, and 4, respectively
- *T1PendantCount, T2PendantCount, T3PendantCount, T4PendantCount*: Number of fixations participants had on the teach pendant for Tasks 1, 2, 3, and 4, respectively
- *T1ObjCount, T2ObjCount, T3ObjCount, T4ObjCount*: Number of fixations participants had on task objects for Tasks 1, 2, 3, and 4, respectively
- *T1InstCount, T2InstCount, T3InstCount, T4InstCount*: Number of fixations participants had on task instruction sheets for Tasks 1, 2, 3, and 4, respectively.
- *T1GripperDur, T2GripperDur, T3GripperDur, T4GripperDur*: Duration of fixations participants had on the robot's gripper in seconds for Tasks 1, 2, 3, and 4, respectively
- *T1BodyDur, T2BodyDur, T3BodyDur, T4BodyDur*: Total duration of fixations participants had on the robot's body (any part of the robot besides the gripper/wrist) in seconds for Tasks 1, 2, 3, and 4, respectively
- *T1PendantDur, T2PendantDur, T3PendantDur, T4PendantDur*: Total duration of fixations participants had on the teach pendant in seconds for Tasks 1, 2, 3, and 4, respectively
- *T1ObjDur, T2ObjDur, T3ObjDur, T4ObjDur*: Total duration of fixations participants had on task objects in seconds for Tasks 1, 2, 3, and 4, respectively
- *T1InstDur, T2InstDur, T3InstDur, T4InstDur*: Total duration of fixations participants had on task instruction sheets in seconds for Tasks 1, 2, 3, and 4, respectively

S6_Dataset.csv

- *UserID*: Participant ID used across all datasets
- *Education*: Highest level of completed education reported by participants
- *Field*: Field of study or employment reported by participants
- *ExpHands*: Participants' level of experience with performing hands-on activities (example: woodworking, crafting) reported on a scale from 1 –5 (1: no experience, 5: lots of experience)
- *ExpRobots*: Participants' level of experience with robots reported on a scale from 1 –5 (1: no experience, 5: lots of experience)
- *ExpTech*: Participants' level of experience with technology reported on a scale from 1 –5 (1: no experience, 5: lots of experience)
- *ExpProg*: Participants' level of experience with programming reported on a scale from 1 –5 (1: no experience, 5: lots of experience)
- *ExpProgRob*: Participants' level of experience with programming robots reported on a scale from 1 –5 (1: no experience, 5: lots of experience)
