# Supplementary material for: Curricula for teaching end-users to kinesthetically program collaborative robots
Source: PLoS One. 2023 Dec 1;18(12):e0294786. doi: 10.1371/journal.pone.0294786 (PMC10691692; doi:10.1371/journal.pone.0294786)
Supplement: S1 Appendix — This appendix includes the questionnaire items we used to measure participants’ confidence with various aspects of programming. (PDF) [file pone.0294786.s010.pdf]

Confidence questionnaire items

Participants were administered pre-study and post-study questionnaires that included the following 5-point Likert scale items measuring their confidence with various aspects of using and programming the UR5 *(1: Strongly Disagree, 5: Strongly Agree)*:

- I am confident that I can successfully program tasks on the robot.
- I feel confident about operating the robot.
- I understand how to move the robot effectively.
- I am uncertain about my ability to program an error-free motion trajectory on the robot.
- I can use the robot's gripper to grip objects effectively.
- I believe I can demonstrate a motion for the robot without significant difficulty.
- I can strategically program the robot to minimize task suboptimalities and failures.
