# Supplementary material for: Curricula for teaching end-users to kinesthetically program collaborative robots
Source: PLoS One. 2023 Dec 1;18(12):e0294786. doi: 10.1371/journal.pone.0294786 (PMC10691692; doi:10.1371/journal.pone.0294786)
Supplement: S2 Appendix — This appendix contains a list of data that was excluded from our analysis. (PDF) [file pone.0294786.s011.pdf]

Excluded study data

The excluded data is listed below (number of participants excluded shown in parentheses with condition, P: practice, C: curriculum):

- **Task 1**: task time, unsuccessful demonstrations *(2C)*; gaze fixation *(1C)*; average force/torque change *(3C, 5P)*; suboptimalities *(3C)*
- **Task 2**: task time, unsuccessful demonstrations, suboptimalities *(1C)*; average force/torque change *(2C, 5P)*
- **Task 3**: average force/torque change *(2C, 5P)*
- **Task 4**: task time, unsuccessful demonstrations *(4C)*; average force/torque change *(5C, 5P)*; gaze fixation *(4C, 2P)*
- **All tasks**: task progress *(4C)*
