# Supplementary material for: Curricula for teaching end-users to kinesthetically program collaborative robots
Source: PLoS One. 2023 Dec 1;18(12):e0294786. doi: 10.1371/journal.pone.0294786 (PMC10691692; doi:10.1371/journal.pone.0294786)
Supplement: S3 Appendix — This appendix lists the models we used for our statistical analysis. (PDF) [file pone.0294786.s012.pdf]

Statistical models

We list the models used for our analysis in the table below. For parametric models, covariates were chosen based on which covariates increased the model's R^2 score. For nonparametric models, Kruskal-Wallis was used for pairwise tests with learning intervention as the independent variable and nonparametric ANCOVA models were used with learning intervention and task as independent variables. For nonparametric ANCOVA models, all covariates were included (gender; age; education level and field; and experience with hands-on activities, technology, robots, computer programming, and programming robots). The learning intervention the participant participated in (practice or curriculum) was used as the independent variable for all models. We used the Shaprio-Wilk test for normality and Levene's test for homoscedasticity.

Dependent Variable	Covariate(s)	Model	Normality	Homoscedasticity
Change in confidence in UR5 programming	None	Kruskal-Wallis	p = .006	N/A
Change in confidence in planning and executing kinesthetic teaching	None	Kruskal-Wallis	p = .009	N/A
Projected confidence	None	Kruskal-Wallis	p < .001	N/A
Adapted NASA TLX	Gender, Education, Prior experience with hands-on activities, robots, technology, and programming robots	ANCOVA	P = .417	P = .528
Task progress	All	Quade Nonparametric ANCOVA	p < .001	N/A
Number of	All	Quade	p < .001	N/A

unsuccessful demonstrations		Nonparametric ANCOVA		
Task time (seconds)	All	Quade Nonparametric ANCOVA	$p < .001$	N/A
Number of program suboptimalities	All	Quade Nonparametric ANCOVA	$p < .001$	N/A
Average rate of change of force (Newtons/sec)	All	Quade Nonparametric ANCOVA	$p < .001$	N/A
Average rate of change of torque (Newton-meters/sec)	All	Quade Nonparametric ANCOVA	$p = .008$	N/A
Gaze fixation duration and quantity on robot gripper	All	Quade Nonparametric ANCOVA	$p < .001$, $p < .001$	N/A
Gaze fixation duration and quantity elsewhere on robot	All	Quade Nonparametric ANCOVA	$p < .001$, $p < .001$	N/A
Gaze fixation duration and quantity on teach pendant	All	Quade Nonparametric ANCOVA	$p < .001$, $p < .001$	N/A
Gaze fixation duration and quantity on task objects	All	Quade Nonparametric ANCOVA	$p < .001$, $p < .001$	N/A
Gaze fixation duration and quantity on task instructions	All	Quade Nonparametric ANCOVA	$p < .001$, $p < .001$	N/A
